# Towards measuring food insecurity stigma: development and validation of the Food Insecurity Self-stigma Scale and the Food Support Experiences Scale

**DOI:** 10.1186/s12889-024-20878-y

**Published:** 2024-11-30

**Authors:** Natalie Taylor, Emma Boyland, Paul Christiansen, Alan Southern, Charlotte A. Hardman

**Affiliations:** 1 Department of Psychology, University of Liverpool, Liverpool, UK; 2 Management School, University of Liverpool, Liverpool, UK

**Keywords:** Food insecurity, Food support, Stigma, Scale development, Psychometric validation

## Abstract

**Background:**

Within high income countries, individuals experiencing food insecurity have become increasingly reliant on food support to satisfy household food needs. However, experiencing food insecurity and accessing food support are highly stigmatised, negatively impacting psychological and emotional wellbeing. Being able to quantify this stigma may contribute towards reducing these impacts. This study aimed to develop and validate two novel scales enabling the quantification of stigma concepts within the food insecurity and food support context: (1) the Food Insecurity Self-stigma Scale (FISS), which measures the level of self-stigma (and related constructs) that individuals experiencing food insecurity feel regarding their food insecure status; and (2) the Food Support Experiences Scale (FSES), which measures the psycho-social experiences (including the experience of self-stigma) when individuals access a food support service.

**Methods:**

English speaking participants who identified as experiencing food insecurity completed the new FISS (*N* = 211) and FSES (*N* = 123) measures, alongside other validation measures. Exploratory (EFA) and confirmatory factor analysis (CFA) were carried out for both scales. Regressions using latent variables derived from the CFA were used to test convergent and divergent validity. McDonald’s Omega was used to assess internal reliability and intra-class correlations between initial and retest FISS and FSES scores of a small number of participants (FISS: *N* = 14; FSES: *N* = 8) were used to assess test-retest reliability.

**Results:**

EFA indicated three-factor structures best fit both scales. CFA revealed a good fit of the model for the FISS (15 items; 3 factors: righteous anger, non-disclosure, and stereotype endorsement). Meanwhile, an acceptable-to-poor fit of the model was revealed for the FSES (23 items; 3 factors: self-approval and disclosure, dietary and interpersonal satisfaction, and perceived effectiveness and impact). Importantly, convergent validity was only found for the non-disclosure subscale of the FISS and the self-approval and disclosure subscale of the FSES.

**Conclusions:**

The FISS and FSES provide valid tools for quantifying aspects of stigma relating to the experience of food insecurity and accessing food support respectively. Development of these two scales may provide an important first step towards measuring stigma. developing interventions which reduce this psychological burden, and working to promote psychological wellbeing within populations experiencing food insecurity.

**Supplementary Information:**

The online version contains supplementary material available at 10.1186/s12889-024-20878-y.

## Background

Recently in the UK, punitive welfare reforms driven by austerity, the COVID-19 pandemic and the subsequent cost of living crisis have made it extremely difficult to afford sufficient food to satisfy household needs, leading to increases in food insecurity [1]. Food insecurity runs on a continuum in which anxiety about food acquisition precedes the experience of not having enough food, with significant mental health impacts across all levels of the continuum [2–4]. Almost one in five households in the UK reported experiencing food insecurity in June 2023 [1], and as a result, more people are relying on alternative ways of accessing food, including food support services such as food banks and food pantries.

Previous research within high income countries has consistently reported that people experiencing food insecurity and those subsequently accessing food support are highly stigmatised. For example, Pineau et al. [5] conducted a narrative review which explored the interrelation between social stigma, social exclusion and food insecurity among women residing in high-income countries. While they found that carrying the status of food insecurity was enough to warrant stigmatisation, for those accessing food support the experience of stigma appeared to be even more pronounced. Consistent with this, an international scoping review of 15 years of qualitative research by Middleton et al. [6] found that stigma as a result of food bank access was present across high-income countries including the USA [7], Canada [8–11], New Zealand [12] and the Netherlands [13]. More recently, in a systematic review conceptualising food banking in the UK [14], 13 of the 24 studies that evidenced psychological implications of food bank use explicitly reported the prevalence of stigma in relation to access. The review also reported a number of other psychological, social and physical health impacts affecting individuals accessing food banks including: experiencing negative emotions such as embarrassment, shame, degradation and powerlessness; an inability to access foods suitable for dietary preferences and needs and to promote health and wellbeing; fear of judgement by staff or the public; and a lack of agency and control. It is likely that these impacts work cumulatively to create a negative and stigmatising experience of accessing food support.

Theoretically, to be stigmatised is to possess an attribute that is ‘deeply discrediting’ [15], which differentiates one from others who do not possess the attribute. This attribute is generally thought of as undesirable [15, 16]. For the stigmatised population, the effects may be largely felt through two mechanisms: public stigma and self-stigma [17, 18]. Public stigma occurs when large numbers of people within society endorse negative stereotypes about the stigmatised population, leading to prejudices such as anger and fear of the group, and discrimination including both obvious and nuanced mistreatment including being ignored, avoided, stared at or patronised [17, 19]. Meanwhile self-stigma refers to the internalisation of public stigma (also known as internalised stigma), resulting in decreased self-esteem and self-efficacy [18]. In cases in which a stigmatised condition may be concealable (or is not outwardly obvious to others), there may also exist a certain level of anticipated stigma, defined as the expectation of being stigmatised should their condition be revealed, due to awareness of related negative stereotypes [20]. Within the wider literature, stigma has been evidenced to have wide-reaching impacts on health, well-being, and life chances [21].

Applying this theoretical perspective to the food insecurity context, over the last decade, a shifting of state responsibility for the effects of austerity (including unemployment and reliance on state welfare) towards the individual has initiated neoliberal discourses of laziness and undeservingness [22–24]. Such discourses trigger negative attitudes towards individuals who are unable to meet societal demands, such as acquiring food to satisfy social norms. These negative attitudes have become normalised into pejorative stereotypes within society (including laziness, lack of education or concern for health, and an inability to implement skills such as budgeting and cooking) with the aid of unsupportive social policy and media misrepresentation of individuals experiencing food insecurity [6, 25–28]. The presence of these negative stereotypes represents public stigma regarding food insecurity and food bank access, leading to prejudice and discrimination against individuals experiencing food insecurity, working as a form of social exclusion and exacerbating inequalities within communities [17, 19]. For example, Psarikidou et al. [29] highlight that individuals accessing food support services such as food banks are often viewed as “receivers” of food, as opposed to “purchasers”, indicating a compounding lack of autonomy and agency, and resulting in reduced social status. Middleton et al. [6] reinforce this through conceptualisation of the receipt of food from food banks as a gift, drawing on Mauss’ [30] theory which states that all gifts are in some way received with an obligation of reciprocation, and to receive a gift with no means or intention to reciprocate presents a threat to social status. Therefore, individuals receiving ‘free’ food from food banks are liable to a hidden cost – a reduction in social status from those who do the gifting, and resulting stigmatisation [6, 31].

The internalisation of negative stereotypes (self-stigma) is arguably hard to measure due to social desirability bias and participants’ lack of awareness of their own implicit attitudes [32, 33]. Yet, reduced self-esteem (an outcome of self-stigma [18]) has been reported within participants experiencing food insecurity and accessing food support across a range of demographics [34–36], indicating the likely prominent role of self-stigma in this context. Additionally, as a result of public stigma, research suggests that individuals experiencing food insecurity may themselves create an ‘Other’ who embodies the negative stereotypes of laziness, undeservingness and incompetence [6, 26, 37], suggesting internalisation of such negative stereotypes (and thus indicating self-stigma). In some cases, this ‘Other’ opposes their own ‘deserving’ situation, thus creating a hierarchy of social status used to stigmatise [6, 26, 37]. This may act as a protection mechanism against their self-stigma and present an outlet for their frustration at their situation. Meanwhile, in other cases, the creation of an ‘Other’ built from negative stereotypes can make worthy individuals feel as if they do not qualify for food support despite their food insecure status [6, 38], a situation which has been reported as increasing hopelessness and frustration by Meza, Altman [39].

In terms of anticipated stigma, firstly, individuals experiencing food insecurity may wish to detach from society and reject other forms of help due to the shame of disclosing their struggles accessing enough food [40, 41]. Secondly, reluctance to access, or return to food support services such as food banks for fear of stigmatisation is highly prevalent [25, 38, 42]. Taken together, this may be particularly problematic, as those attending food banks may be already consuming a diet lacking in nutritional quality and quantity [43, 44]. Therefore, non-participation in food support may further diminish the nutritional load of the diet, pushing individuals into micronutrient (and potentially macronutrient) deficiency, which has significant physical health impacts.

Hence, it is clear that stigma within populations experiencing food insecurity must be addressed. Yet, quantitative data surrounding the issue are lacking, and to our knowledge, no scales have been developed and validated which measure experiences of stigma in populations with food insecurity. Kindle et al. [45] developed the Food Pantry Stigma Scale through adaptation of the HIV Stigma Scale [46]. This study was based in America, with the term ‘food pantries’ here referring to the model commonly known as ‘food banks’ in the UK. The scale followed the theoretical perspective that increased stigma is reflected by increases in desired social distance from the relevant group. While avoidance is an important negative response which follows the endorsement of stereotypes and stigmatising attitudes [47], food insecurity is not a condition which is conceived to be contagious or dangerous, therefore, social distance may not be the most salient factor in stigmatisation of this group. Additionally, the Food Pantry Stigma Scale has not yet been validated for use, and since the study was conducted in 2019, societal attitudes towards individuals experiencing food insecurity may have significantly changed and awareness of food insecurity significantly increased. Meanwhile the 24-item Paradox of Self-Stigma scale (PaSS-24) offers a useful tool to measure self-stigma in individuals with mental illness. The PaSS-24 [48] was based upon Corrigan’s social-cognitive model of internalised stigma (otherwise known as self-stigma), which emphasises the role of paradoxical empowerment [17, 18, 48, 49] and shows good psychometric properties. It measures self-stigma through three constructs which are theoretically linked to self-stigma development and maintenance [18, 48]: stereotype endorsement (cognitive level; the degree to which respondents agree with common stereotypes – e.g. ‘People with my condition should be banned from certain jobs’), non-disclosure (behavioural level; trying to hide the possession of a stigmatising characteristic from others – e.g. ‘Because of people’s preconceptions, I do not speak to anybody about the problems linked to my condition’), and righteous anger (paradoxical element; a legitimate level of anger in response to the stigma surrounding the characteristic – e.g. ‘I am angry about the way my condition is caricatured on television’). Corrigan’s [17, 18, 49] model was developed with individuals experiencing various mental health conditions and upon validation of the PaSS-24, the scale was recommended for validation and use within other stigmatised groups [48]. The PaSS-24 scale may have transferability to the context of food insecurity self-stigma, as the endorsement of negative stereotypes about people experiencing food insecurity [6, 26, 37], non-disclosure of low food security status or need to access food support [26, 50–52] and anger and frustration about their food situation and lack of ability to change it [39, 53–55] have all been observed within individuals experiencing food insecurity in previous research.

Therefore, the aim of this study was to provide a starting point for quantifying concepts of stigma relating to food insecurity and food support access by developing and validating two novel measures. It was deemed appropriate to start this quantification with measuring self-stigma, due to the obvious presence of public stigma, and the significant potential mental health effects of self-stigma, including loss of self-esteem [18]. The first measure, the Food Insecurity Self-stigma Scale (FISS), aimed to measure self-stigma and related constructs in individuals experiencing food insecurity through adaptation of the PaSS-24 to the food insecurity context. The second measure, the Food Support Experience Scale (FSES), aimed to measure the psycho-social experience (including the experience of self-stigma) of accessing food from a food support provider, which may be a food bank, food pantry or other type of food support. This includes items measuring self-stigma as a person who accesses food support, alongside other psychological, social and practical aspects of receiving food from the support provider that may contribute to overall experience. There is important nuance between the two measures, with the FISS measuring only self-stigma in people experiencing food insecurity, who may not necessarily be accessing food support, while the FSES measures self-stigma, alongside other aspects, when accessing food support specifically. Developing two separate scales is important for two reasons. Firstly, not all individuals experiencing food insecurity access food support [56, 57], therefore it is important to be able to measure the stigma that this group feel in relation to their food insecurity status. Secondly, for those who do access food support, it is not clear whether the stigma experienced is due to being food insecure per se, or manifests as a result of accessing food support. Therefore, developing measures of self-stigma and related constructs for food insecurity and the overall experience of accessing food support may help to provide important quantitative evidence for stigmatization of individuals experiencing food insecurity and/or accessing food support. In turn, these measures may extend existing measures of food insecurity and contribute towards the development of interventions which focus on promoting health and wellbeing in this vulnerable population.

## Methods

### Participants (recruitment and eligibility)

The sample size was based on recommendations for between 5 and 10 participants per scale item, as is considered appropriate for factor analysis in the context of scale development [58]. As individuals experiencing food insecurity are known to be a particularly hard to recruit population, it was considered that five participants per scale item would be sufficient. Therefore, we aimed to recruit 90 participants onto the FISS (18 items), and 115 participants onto the FSES (23 items). Participants were largely recruited via public advertisements on Facebook, however, the advertisement was also shared on Twitter (now known as X). Small amounts of recruitment also took place at local food support services using printed fliers, and via the University of Liverpool School of Psychology Experiment Participation Requirement (EPR) scheme, in which first year students are obliged to earn points by volunteering as participants in research being conducted at the university. The study was advertised as a ‘Food Access and Emotions Study’. Individuals over the age of 18 years, living in the UK and fluent in English were eligible to take part. Participants also needed to be experiencing moderate to severe food insecurity, which was ascertained through a screening question at the start of the questionnaire. A secondary screening question was also included after completion of the FISS, which allowed continuation onto the FSES only if participants had accessed food support in the last six months (see Sect. 2.2.1 below for more details on screening questions). Participants were reimbursed for their time through voluntary entry into a prize draw for £50 supermarket vouchers and/or course credit (through the University of Liverpool School of Psychology EPR scheme). Recruitment took place between November 2022 and June 2023, with this large window reflective of the difficulty in recruiting the required population.

### Measures

#### Screening questions

Participants were required to complete an initial online screening questionnaire in order to verify their food insecurity status. This screening utilised three questions which have been previously adapted from the United States Department of Agriculture’s Adult Household Food Security Survey Module (USDA AFSSM) [59] by the Food Foundation, a UK thinktank which conducts regular measuring and reporting of food insecurity in the UK through their Food Insecurity Tracker [1]. The three screening questions measure moderate to severe food insecurity within the context of high-income countries. As the measure is only three items, its implementation helped to minimise participant burden. Participants were asked to report whether, in the last six months, themselves or anyone in their household had: [1] ‘had smaller meals than usual or skipped meals [2], ever been hungry but not eaten, and [3] not eaten for a whole day, because they ‘could not afford, or get access to food’. This enabled participation by people who were experiencing food insecurity due to a number of causes (e.g. low income, geographical lack of access to supermarkets and/or food support, physical lack of access to supermarkets and/or food support). Answering ‘Yes’ to at least one of these three questions indicated some level of moderate to severe food insecurity, enabling participants to proceed onto answering the FISS and FSES items. Answering ‘No’ to all three questions automatically terminated the study, and participants were directed to the Participant Debrief and the option to enter a voluntary prize draw, for the opportunity to win £50 supermarket vouchers as a reimbursement for their time.

As the FSES measures psycho-social experiences as a result of accessing food support, a secondary screening question was placed before completion of the FSES. This asked whether participants had, in the last six months, ‘received food support from a food support provider such as a food bank, food pantry or community food store, hot meal provider (e.g. a soup kitchen), or other form of food support’. Answering ‘Yes’ to this question confirmed food support access, enabling participants to proceed onto answering the FSES. If participants answered ‘No’, they were not eligible to complete the FSES items, and so were directed to the next set of measures.

#### Food Insecurity Self-stigma Scale (FISS)

**Fig. 1:**
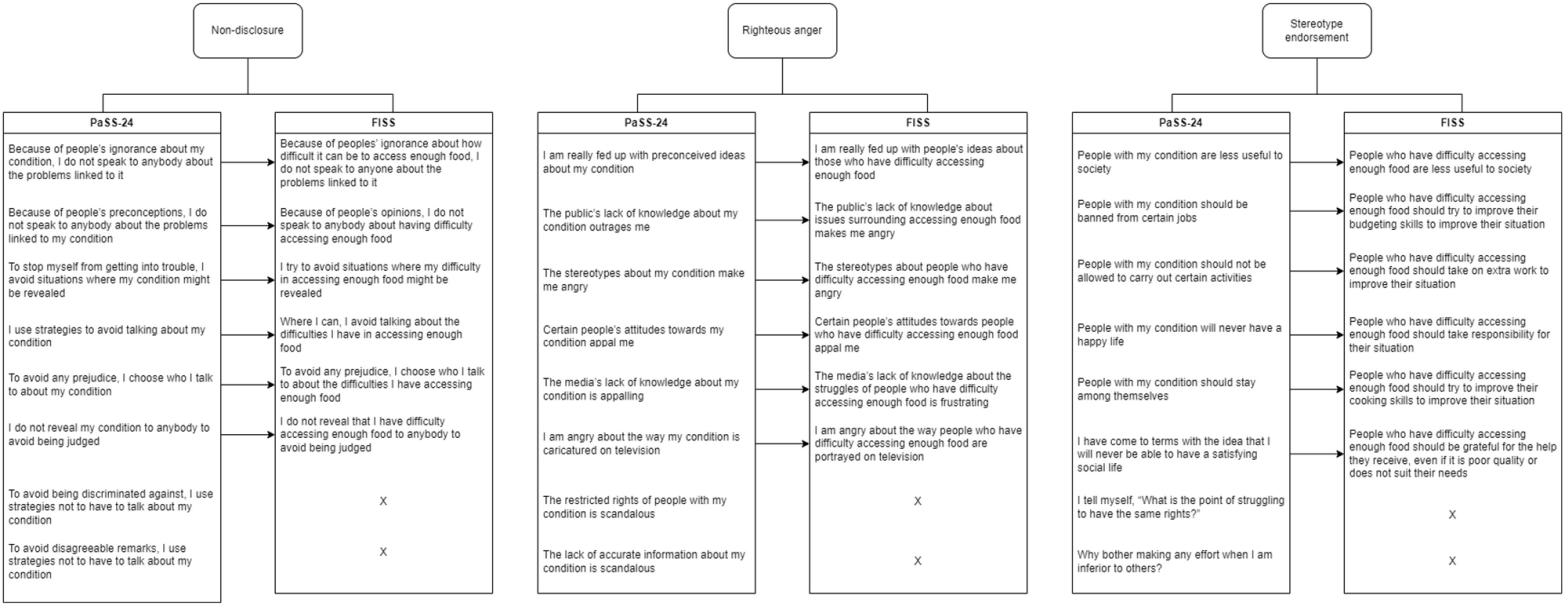
Transforming items on the PaSS-24 scale to suit the food insecurity context for use in the FISS

An 18-item, 5-point Likert scale (‘Strongly disagree’ to ‘Strongly agree’) measuring aspects of self-stigma (stereotype endorsement, non-disclosure and righteous anger) regarding food insecurity was adapted from the Paradox of Self-Stigma scale (PaSS-24) developed and validated by Golay et al. [48] (see Fig. 1). Selection of the PaSS-24 scale for adaptation to the food insecurity context was based on theoretical fit and also on feedback from stakeholders working directly with individuals experiencing food insecurity, which emphasised that any scale used to measure self-stigma within populations experiencing food insecurity should be indirect and impartial where possible, so as not to further exacerbate self-stigma in participants. The PaSS-24 was felt to be a useful tool in this respect because several items are worded in relation to third parties which allows participants to provide responses which indicate their biases towards other people with their stigmatised condition (e.g. by phrasing items as ‘People who have difficulty accessing food are less useful to society’ as opposed to ‘I am less useful to society’). This may therefore aid in indirectly measuring the internalisation of negative stereotypes about people experiencing food insecurity [17, 18], without contributing to further self-stigma. This was important as previous self-stigma scales in other contexts have been deemed insensitive, resulting in non-completion by participants and limiting their practical utility [49]. Additionally, the righteous anger element from the PaSS-24 scale was deemed an appropriate fit with food insecurity by stakeholders because it allowed participants to embrace their paradoxical empowerment, potentially helping to moderate and minimise potential negative mental health impacts of completing the survey. The PaSS-24 has strong psychometric properties and is validated to measure self-stigma relating to mental illness, with the authors suggesting its transferability to other stigmatised conditions and contexts [48].

Items within the non-disclosure and righteous anger subscales were easily adapted to suit the food insecurity context by modifying the wording. For example, items in the PaSS-24 referring to a stigmatised ‘condition’ were reworded, defining the ‘condition’ as having ‘difficulty accessing enough food’. Wording was also adapted in certain cases to make the questions easy to comprehend (e.g. ‘people’s preconceptions’ was replaced with ‘people’s opinions’). Meanwhile, stereotypes of individuals suffering from a mental health condition, as reflected in the items under the stereotype endorsement factor, are different from stereotypes of individuals experiencing food insecurity. Therefore, items in the PaSS-24 classed under the stereotype endorsement factor were adapted to commonly held stereotypes of individuals experiencing food insecurity, including laziness and aversion to work, poor personal choices, a lack of budgeting and cooking skills, and a societal expectation of gratitude for any and all support, despite its quality [23, 25, 37, 60, 61]. Items in the original PaSS-24 which were unrelated to the food insecurity context (e.g. ‘The restricted rights of people with my condition is scandalous’), seemed repetitive (e.g. ‘To avoid disagreeable remarks, I use strategies not to have to talk about my condition’), or were overly negative or stigmatising themselves (e.g. ‘Why bother making any effort when I am inferior to others?’) were not adapted and were excluded. This included two items from each of the three factors from the original version of the PaSS-24 and so an even split of items across the three factors was maintained for analysis. As stated previously, item adaptation and removal of overly negative items was done in response to feedback from external stakeholders in order to make the scale as relevant as possible, and restrict any further negative feeling as a result of completion. Items were then ordered to follow a repeating cycle of non-disclosure, righteous anger, then stereotype endorsement. Higher scores on the FISS indicate higher levels of self-stigma as a result of being food insecure.

#### Food Support Experiences Scale (FSES)

This scale is designed to measure psycho-social experiences of food support access, including the experience of self-stigma and other social, psychological and practical aspects. A 23-item, 5-point Likert scale (‘Strongly disagree’ to ‘Strongly agree’) was developed from previous qualitative studies with participants experiencing food insecurity and/or those accessing food support services [2–4, 23, 25, 26, 28, 37, 38, 50, 60, 62–69]. Domains implicated as playing a role in the process of stigmatisation and contributing to overall negative experience of accessing food support were identified. This qualitative research was largely based in the UK, and is synthesised and summarised in Taylor et al. [14] but also includes evidence from a review of qualitative studies from high income countries [6]. As outlined previously, a second screening question was required which ensured participants only had access to this questionnaire if they had ‘received food support from a food support provider such as a food bank, food pantry or community food store, hot meal provider (e.g. a soup kitchen), or other form of food support’ within the last six months. This wording was used to enable relevance to a range of food support services, not only charitable services. For example, this may include people’s experiences of accessing food from social businesses where food is paid for (albeit at a reduced price), which may be perceived as a potentially more socially acceptable way of acquiring food. Participants were also required to indicate their main food provider and respond with their access to this provider only in mind. Higher total scores on the FSES indicate lower levels of self-stigma and a more positive overall experience when accessing food support.

#### Assessment of convergent and divergent validity

Both the Rosenberg Self-Esteem (RSE) scale and the Depression, Anxiety and Stress (DASS-21) scale were used to assess convergent validity. The RSE scale is a validated 10-item, 4-point Likert scale (‘Strongly disagree’ to ‘Strongly agree’) measuring self-worth and self-esteem within participants [70]. A total RSE score was calculated (reliability scores: FISS w_t_ = 0.91; FSES w_t_ = 0.92) with higher scores on the RSE scale indicating higher self-reported self-esteem. Self-esteem is considered to be inversely related to stigma [71, 72], therefore the RSE scale was considered appropriate for assessing convergent validity. Scores on the FISS were expected to be negatively correlated with RSE scale scores and scores on the FSES were expected to be positively correlated with RSE score.

The DASS-21 scale [73] was used to identify the presence of depression, anxiety and stress in participants, using a 4-point Likert scale (‘Never’ to ‘Almost always’). The 21-item version was selected over the 42-item version in order to minimise participant burden. Individual scores were calculated across each of the three subscales: depression (reliability scores: FISS w_t_ = 0.94; FSES w_t_ = 0.95), anxiety (reliability scores: FISS w_t_ = 0.90; FSES w_t_ = 0.91), and stress (reliability scores: FISS w_t_ = 0.89; FSES w_t_ = 0.90). Higher scores on the corresponding subscale indicate higher levels of depression, anxiety and stress. Stigma relating to food support access has been previously associated with negative mental health outcomes including depression, anxiety and stress. Therefore, higher scores on each DASS-21 subscale were expected to correlate positively to FISS scores and correlate negatively with FSES scores [74, 75].

Meanwhile, the International Physical Activity Questionnaire - Short Form (IPAQ-SF) was used to assess divergent validity. The IPAQ-SF is a 7-item, open ended questionnaire measuring physical activity across 3 levels (vigorous, moderate or walking), conducted in the previous 7 days [76]. Scores are calculated for each of the three levels by multiplying the time spent doing physical activity by the number of days on which the activity was conducted. These scores are then weighted using MET levels (walking = 3.3 METs, moderate intensity = 4 METs, vigorous intensity = 8 METs) and summed to get a final (total) score (reliability scores: FISS w_t_ = 0.74; FSES w_t_ = 0.81). Again, the short-from version was selected in order to minimise participant burden. It was expected that level of physical activity would not correlate with FISS or FSES scores.

### Procedure

#### Ethical approval

was gained for the study from University of Liverpool’s Research Ethics Committee (reference number 11315). The study protocol and analysis strategy were pre-registered on the Open Science Framework before data collection commenced [77].

Survey items for both the FISS and FSES were drafted and then sent to relevant stakeholders currently working with individuals who are experiencing food insecurity for preliminary checking and feedback. Changes were made to survey items to address this feedback, including adaptation of language to better suit the study population (e.g. changing ‘food insecurity’ to ‘difficulty accessing enough food’ in FISS items) and to promote a more positive framing (for example swapping ‘Getting food from my food support provider is an undignified experience’ for ‘Getting food from my food support provider is a dignified experience’ in FSES items), thus explaining why higher scores on the FSES indicate a more positive overall experience (including less self-stigmatisation) of accessing food support. Considering the positionality of the research team as outsiders to the phenomenon in question, this changing of language and framing is highly important and relevant so as not to further marginalise the population [78].

The survey items were then uploaded into a single survey on Qualtrics.com, for participants to complete online. Participants were required to first read the participant information sheet and consent to the study, before generating a unique 7-digit ID code which could be used to link survey data in order for test-retest reliability to be established. Participants were asked to formulate their unique ID code from the first two letters of their first name, followed by the first three letters of the month they were born in, and finally the last two letters of their surname. Participants were then asked to answer the initial 3-item food insecurity screening question to ensure they were experiencing moderate to severe food insecurity, as per the eligibility criteria. If participants identified as experiencing moderate to severe food insecurity, they were then asked to provide some demographic data relating to their gender, age, ethnicity, income, education and employment status, living situation, and access to food support (including type of food support accessed, and frequency and format of access). All demographic questions were closed, multiple choice questions, apart from age, which had a validated response format, meaning that participants were limited to entering just two characters. Where appropriate, participants had the option to respond to the demographic questions using free text if they selected the option ‘Other – please specify’. Initially, participants completed the FISS, RSE, DASS-21, and IPAQ-SF measures, followed by the FSES. However, due to the length of the overall survey and the resulting level of non-completion, it became apparent that the FSES should be accessed earlier in the questionnaire, to ensure maximum responses. Subsequently, participants completed the FISS, then the FSES, followed by the measures for convergent and divergent validity. At the point of completion of the FSES, the secondary screening question was implemented, and participants who did not identify as having accessed food support within the last six months were directed to the end of the block, skipping the FSES and moving on to complete the measures for convergent and divergent validity.

Once the full questionnaire was completed, participants were directed to the participant debrief, and given the opportunity to enter the prize draw. In order to ascertain test-retest reliability, a sample of participants completed the survey again after between two and nine weeks. The long period included for test-retest again reflects the difficulty in recruiting the relevant population.

### Data analysis

Data were analysed with R version 4.3.1, using the ‘lavaan’ and ‘psych’ packages. The FISS and FSES were analysed separately, as they are separate scales, yet the same analysis strategy was used on both scales.

#### Data preparation

Prior to analyses, responses on the FISS and FSES were assigned a value of 1 to 5 (1 = strongly disagree, 2 = somewhat disagree, 3 = neither agree nor disagree, 4 = somewhat agree, 5 = strongly agree). Higher scores on the FISS indicated higher levels of self-stigma, while higher scores on the FSES indicated a more positive overall experience (including less self-stigmatisation) of food support access. Four items on the FSES were reverse scored in order for inter-correlations to remain positive between scale items. No items on the FISS required reverse scoring. Sampling adequacy was checked on both scales using the Kaiser-Meyer-Olkin (KMO) statistic and Bartlett’s Test for Sphericity was used to assess whether correlations were sufficient to run factor analysis (*p* < 0.01). Each dataset was randomly split into 2 groups for exploratory factor analysis (EFA datasets: FISS *n* = 109; FSES *n* = 62) and confirmatory factor analysis (CFA datasets: FISS *n* = 102; FSES *n* = 61).

#### Exploratory factor analysis

Parallel analysis was used to estimate the number of factors within each dataset. Exploratory factor analyses with an oblique rotation were then conducted on the EFA datasets, as factors were expected to correlate with each other. Items with factor loadings < 0.4 were discarded [79], as were those that cross-loaded, namely had two or more factor loadings of > 0.32 [80].

#### Confirmatory factor analysis

Confirmatory factor analyses were subsequently performed using a maximum likelihood with Satorra Bentler correction (MLM) fitting method. The fit of the model was assessed using the Comparative Fit Index (CFI), the Tucker-Lewis Index (TLI), the Root Mean Square of Approximation (RMSEA), and the Standardised Root Mean Square Residual (SRMR). The values for the CFI and TLI were considered good if above 0.95 and acceptable if above 0.90 [81]. The RMSEA indicated a good fit for the model if the score was < 0.06 and a moderate but acceptable fit if the score lay between 0.06 and 0.08 [81]. The SRMR indicated a good fit if the score was < 0.08 [81]. In the case that the model fit could be improved, modification indices were examined and covariance pathways between residuals were added if items were on the same subscale and the correlated residuals made conceptual sense.

#### Convergent and divergent validity

Regressions using latent variables derived from the confirmatory factor analysis were used to assess convergent and divergent validity of the FISS and FSES compared with the RSE, DASS-21, and IPAQ-SF.

#### Internal reliability

McDonald’s Omega was used to assess internal reliability for both the FISS and FSES, with values acceptable if above 0.7 [82]. Omega Total was calculated for each observed subscale. Omega Hierarchical was also computed for the full scales to explore the extent to which variance attributed to a common factor (’g’) was reliable. This allowed us to ascertain whether a total score of the scale can be reliably used, or whether scoring of individual subscales would be more appropriate.

#### Test-retest reliability

Test-retest reliability was assessed through examination of the intra-class correlation between initial and retest FISS and FSES scores. Intra-class correlations of 0.60 or higher were considered to show good test-retest reliability [83].

## Results

The online survey achieved 385 responses, yet not all responses were usable due to failure to complete all the FISS and/or FSES items, resulting in incomplete data. A total of 211 participants completed the FISS and were then eligible to continue on to complete the FSES, having passed the food support access screening question. Of these, 123 participants subsequently completed the FSES scale. Participation exceeded the acceptable level of five participants per scale item for both the FISS (18 items; acceptable *n* = 90) and the FSES (23 items; acceptable *n* = 115) [58]. Table 1 shows the demographic characteristics of participants who completed the FISS and FSES scales.

**Table 1:** Demographic characteristics of participants taking part in the FISS and FSES

	FISS (*n* = 211)	FSES (*n* = 123)
Gender:		
Female	165	97
Male	27	15
Non-binary / third gender	16	9
Prefer not to say	3	2
**Age (years)**:		
Mean (s.d.)	40.43 (± 13.00)	41.74 (± 11.91)
Range	18–70	18–70
**Ethnicity**:		
White British	150	83
White Irish	6	3
Other White background	18	10
Black - Caribbean	5	4
Black – African	5	3
Other Black background	2	2
Asian – Indian	4	2
Asian – Pakistani	7	6
Other Asian background	4	4
Mixed – White and Black Caribbean	0	3
Mixed – Black and White African	4	0
Other mixed background	4	1
Chinese	1	1
Prefer not to say	1	1
**Household composition**:		
Living alone	54	33
Partner (married/unmarried)	77	42
Children	93	60
Parents	18	14
Siblings	12	7
Extended family	7	4
Housemates	22	7
**Education**:		
No formal qualifications	11	8
GCSE or equivalent	30	22
BTEC/NVQ/Diploma	42	31
A level or equivalent	36	14
University degree	85	43
Other	7	5
**Employment status**:		
Full-time employed	48	21
Part-time employed	41	24
Not employed for pay	28	22
Caregiver	38	30
Full-time student	27	7
Part-time student	10	4
Retired	12	7
Other	33	22
**Net household income**:		
< £5,200	24	18
£5,200 - £10,399	26	19
£10,400 - £15,599	33	19
£15,600 - £20,799	36	24
£20,800 - £25,999	27	16
£26,000 - £36,399	17	11
£36,400 - £51,999	15	8
£52,000 - £77,999	7	3
> £77,999	3	0
Not sure/don’t know	23	5
**Type of food support accessed**:		
Food bank Food pantry/community food store	59 81	55 71
Hot meal provider	32	28
Other	25	18
None/not applicable	76	8
**Regularity of food support access (if accessed)**:		
Weekly	43	40
Fortnightly	21	18
Once a month	31	22
As and when needed	37	30
Other	6	5
Not applicable	73	8
**Format of food support access (if accessed)**:		
Home delivery	22	17
Attend the food support provider in person	95	84
Someone else attends the food support provider for me	16	10
Other	5	4
Not applicable	73	8

Examining the demographic data, it was noted that 10 participants reported a household income of £52,000 or above, thus raising doubt over the food insecurity status of these participants. These high incomes could not be explained by a higher number of earners living in the house and hence these 10 responses were removed, still maintaining over five participants per scale item in each of the FISS and FSES scales [58]. This sensitivity analysis occurred after the data for the FISS and FSES scales had been split into EFA and CFA datasets. Therefore, the responses of the 10 participants reporting a high income were removed from FISS EFA (*n* = 5) and CFA (*n* = 5) datasets by cross referencing with the whole dataset. The new FISS EFA (*n* = 104) and CFA (*n* = 97) datasets were used in the analysis. Similarly, of the 10 participants reporting high income, three completed the FSES scale. Their responses were also removed from FSES EFA (*n* = 1) and CFA (*n* = 2) datasets by cross referencing with the whole dataset. The new FSES EFA (*n* = 61) and CFA (*n* = 59) datasets were then used for the analysis.

### Food Insecurity Self-stigma Scale (FISS)

#### Data preparation

The KMO statistic exceeded the acceptable level of 0.7 (KMO = 0.82), and Bartlett’s Test for Sphericity indicated that correlations between items were large enough for factor analysis (X^2^(153) = 950.19; *p* < 0.001).

#### Exploratory factor analysis

The parallel analysis estimated that a three-factor solution may be most applicable to the data. Applying this, two-, three- and four-factor solutions were tested, with the factor analyses revealing that a clear three-factor solution best suited the data, according to factor loadings. The three-factor solution explained 52% of variance within the 18 items. Factor one explained 21% of variance; Eigenvalue = 3.83. Factor two explained 21% of variance, Eigenvalue = 3.76. Factor three explained 9% of variance; Eigenvalue = 1.71. Table 2 provides the item-factor loadings. Items three (‘People who have difficulty accessing enough food should take responsibility for their situation’), six (‘People who have difficulty accessing enough food are less useful to society’), and 18 (‘People who have difficulty accessing enough food should be grateful for the help they receive, even if it is poor quality or does not suit their needs’) did not load onto any factor sufficiently (see Table 2), and were therefore discarded. All other items loaded onto one factor respectively, without any cross-loading (as defined in Sect. 2.4.2). Factors one and two were made up of six items each, and factor three was made up of three items, following the discarding of the three items. The three factors followed the factor structure suggested by Golay et al. [48] in the PaSS-24 scale as expected. Factor one comprised items concerning righteous anger (for example, ‘I am really fed up with people’s ideas about those who have difficulty accessing enough food’). Factor two comprised items referring to non-disclosure of food insecurity status (for example, ‘I try to avoid situations where my difficulty in accessing enough food might be revealed’). Factor three comprised items relating to stereotype endorsement (for example, ‘People who have difficulty accessing enough food should try to improve their budgeting skills to improve their situation’).

**Table 2:** FISS item factor loadings items that were removed are shown in italics

Item		Factor 1	Factor 2	Factor 3
		Righteous anger	Non-disclosure	Stereotype endorsement
1	Because of peoples’ ignorance about how difficult it can be to access enough food, I do not speak to anyone about the problems linked to it	-0.01	**0.81**	0.14
2	I am really fed up with people’s ideas about those who have difficulty accessing enough food	**0.72**	0.07	0.09
*3*	*People who have difficulty accessing enough food should take responsibility for their situation*	*-0.29*	*0.13*	*0.39*
4	Because of people’s opinions, I do not speak to anybody about having difficulty accessing enough food	0.04	**0.76**	0.02
5	The public’s lack of knowledge about issues surrounding accessing enough food makes me angry	**0.76**	-0.13	-0.10
*6*	*People who have difficulty accessing enough food are less useful to society*	*-0.38*	*-0.14*	*0.20*
7	I try to avoid situations where my difficulty in accessing enough food might be revealed	-0.04	**0.82**	-0.04
8	The stereotypes about people who have difficulty accessing enough food make me angry	**0.71**	0.14	-0.07
9	People who have difficulty accessing enough food should take on extra work to improve their situation	-0.41	0.06	**0.41**
10	Where I can, I avoid talking about the difficulties I have in accessing enough food	0.10	**0.77**	-0.06
11	The media’s lack of knowledge about the struggles of people who have difficulty accessing enough food is frustrating	**0.77**	0.05	0.05
12	People who have difficulty accessing enough food should try to improve their cooking skills to improve their situation	0.07	-0.05	**0.69**
13	To avoid any prejudice, I choose who I talk to about the difficulties I have accessing enough food	0.12	**0.67**	0.00
14	I am angry about the way people who have difficulty accessing enough food are portrayed on television	**0.72**	0.04	0.10
15	People who have difficulty accessing enough food should try to improve their budgeting skills to improve their situation	0.01	0.01	**0.79**
16	I do not reveal that I have difficulty accessing enough food to anybody to avoid being judged	-0.05	**0.78**	-0.08
17	Certain people’s attitudes towards people who have difficulty accessing enough food appal me	**0.71**	0.03	0.00
*18*	*People who have difficulty accessing enough food should be grateful for the help they receive*,* even if it is poor quality or does not suit their needs*	*-0.03*	*-0.11*	*0.34*

Factor loadings shown in bold highlight which factor onto which the respective item loaded

#### Confirmatory factor analysis

**Fig. 2:**
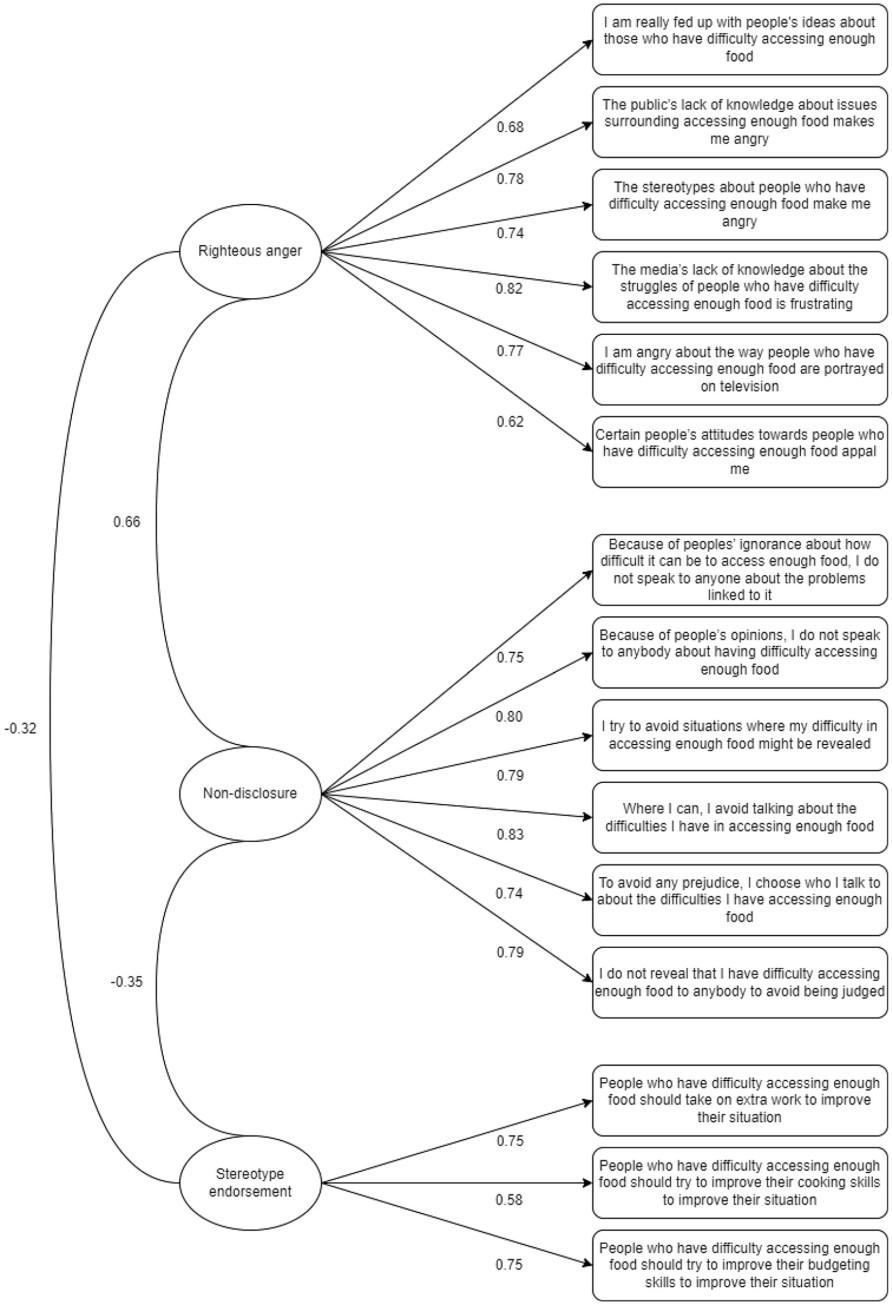
FISS factor model with standardised factor loadings (single-headed arrows) and covariances (double headed arrows)

Six items were free to load onto the latent factors of righteous anger and non-disclosure respectively, and three items were free to load onto the latent factor stereotype endorsement. The confirmatory factor analysis suggested a good fit of the model to the data (CFI = 0.963; TLI = 0.955; RMSEA = 0.054; SRMR = 0.078), without the need for additional modification indices to be used. Standardised factor loadings were all significant (*p* < 0.001) indicating that all items reflected their underlying latent variable appropriately. The resulting 15-item FISS with scoring instructions is provided in the supplementary material. Figure 2 shows the FISS factor model with standardised factor loadings (single-headed arrows) and covariances (double headed arrows). Unlike in the PaSS-24 scale, where all three factors were positively correlated [48], in the FISS scale, only the factors non-disclosure and righteous anger were positively correlated, while correlations between the other factors were negative.

#### Internal reliability

Factor analysis demonstrated that the FISS is made up of three subscales (non-disclosure, righteous anger, and stereotype endorsement). The McDonald’s Omega hierarchical value for the full scale was below the acceptable value of 0.7 (w_h_ = 0.67), suggesting that there is not reliable variance in responses produced by a common factor across all sections. Reliability statistics were produced for each subscale and all achieved acceptable estimates (righteous anger w_t_ = 0.90; non-disclosure w_t_ = 0.93; stereotype endorsement w_t_ = 0.73). Taken together, this suggests that FISS scores are reliable across individual subscales, but a reliable total scale score cannot be produced.

#### Convergent validity

Regressions using latent variables derived from the confirmatory factor analysis showed that the RSE was only significantly associated (unstandardised coefficient = -0.234; s.e. = 0.084; 95% CI = -0.389, -0.069) with items on the non-disclosure subscale, being in the expected negative direction. Associations between the RSE and the remaining two subscales were non-significant. Using scale means as opposed to latent variables, the overall model was significant, explaining 12% of variance in RSE scores (adjusted R^2^ = 0.12, F(3,195) = 10.01, *p* < 0.001). Again, a significant association was only found with items on the non-disclosure subscale (unstandardised coefficient = -0.325; s.e. = 0.082; 95% CI = -0.487, -0.164; *p* < 0.001), with non-significant associations between RSE scores and stereotype endorsement or righteous anger.

Similarly, regressions using latent variables derived from the confirmatory factor analysis showed that depression (unstandardised coefficient = 0.322; s.e. = 0.078, 95% CI = 0.170, 0.474) anxiety (unstandardised coefficient = 0.160; s.e. = 0.045, 95% CI = 0.07, 0.248) and stress (unstandardised coefficient = 0.206; s.e. = 0.058, 95% CI = 0.093, 0.320) all significantly positively correlated with items on the non-disclosure subscale, but correlations with the other two subscales were non-significant. Using scale means as opposed to latent variables, the overall models were all significant (depression: 11% of variance, adjusted R^2^ = 0.105, F(3, 194) = 8.67, *p* < 0.001; anxiety: 5% of variance, adjusted R^2^ = 0.046, F(3, 194) = 4.134, *p* = 0.007; stress: 11% variance, adjusted R^2^ = 0.111, F(3, 194) = 9.18, *p* < 0.001). Regressions using scale means also showed that depression (unstandardised coefficient = 0.350; s.e. = 0.077; 95% CI = 0.198, 0.503; *p* < 0.001) anxiety (unstandardised coefficient = 0.231; s.e. = 0.070; 95% CI = 0.094, 0.368; *p* = 0.001) and stress (unstandardised coefficient = 0.242; s.e. = 0.064; 95% CI = 0.115, 0.368; *p* < 0.001) all significantly positively correlated with items on the non-disclosure subscale, but correlations with the other two subscales were non-significant.

#### Divergent validity

Regressions using the latent variables indicated that IPAQ-SF total scores were significantly correlated to righteous anger (unstandardised coefficient = 1215.564; s.e. = 530.567, 95% CI = 175.672, 2255.456) and non-disclosure (unstandardised coefficient = -1101.833; s.e. = 509.227, 95% CI = -2099.899, -103.766) but not significantly associated to stereotype endorsement. Using scale means as opposed to latent variables, the overall model was significant, explaining 11% of variance in IPAQ scores (adjusted R^2^ = 0.11, F(3,109) = 5.51, *p* = 0.001). Regressions using scale means indicated that IPAQ-SF total scores were significantly correlated to righteous anger (unstandardised coefficient = 171.08; s.e. = 50.23; 95% CI = 71.52, 270.64; *p* < 0.001) and non-disclosure (unstandardised coefficient = -150.95; s.e. = 46.60; 95% CI = -243.316, -58.591; *p* = 0.002) but also significantly associated to stereotype endorsement (unstandardised coefficient = 125.29; s.e. = 54.78; 95% CI = 16.711, 233.870; *p* = 0.024). Significant correlations found between scores on the FISS and IPAQ-SF indicate poor divergent validity.

#### Test-retest reliability

Due to difficulties in recruitment, recruiting onto the retest presented a further challenge. As a result, the time frame for retest was 9 weeks, in order to recruit as many individuals as possible. We obtained 19 retest participants for the FISS, but the data for only 14 of these could be linked using the unique ID codes provided in test one and test two. None of the retest cohort were excluded due to reporting high income. Mean scores for the initial test and retest were calculated and test-retest reliability was found to be good (ICC = 0.73).

### Food Support Experiences Scale (FSES)

#### Data preparation

The KMO statistic was acceptable (KMO = 0.81) and correlations between items were sufficiently large for carrying out factor analysis (X^2^(253) = 1053.65; *p* < 0.001).

#### Exploratory factor analysis

The new EFA dataset for the FSES (*n* = 61) was constructed for use in conducting an exploratory factor analysis. Parallel analysis using the FSES dataset revealed that a two-factor solution may fit the data best. Two-, three- and four- factor solutions were tested, and items falling into each factor were examined and compared for similarity and relevance to each other. Considering the grouping of items into factors and their conceptual representations, the three-factor solution appeared to be the best fit for the FSES items, explaining 60% of the variance across the 23 items. Factor one was responsible for 24% of the variance; Eigenvalue = 5.48, factor two explained 21% of the variance; Eigenvalue = 4.83, and factor three explained 15% of the variance; Eigenvalue = 3.38. Items 5 (‘Getting food from my food support provider makes me feel in control’), 11 (‘Getting food from my food support provider is a dignified experience’) and 14 (‘I feel equal to others when I get food from my food support provider’) cross loaded over two factors and item 12 (‘I feel indebted to my food support provider when I get food from them’) did not have a sufficient factor loading for any of the factors, therefore these items were discarded. Table 3 displays the factor loadings for the three-factor structure observed.

**Table 3:** FSES item factor loadings

Item		Factor 1	Factor 2	Factor 3
		Self-approval and disclosure	Dietary and interpersonal satisfaction	Perceived effectiveness and impact
1	It is easy for me to get foods which satisfy my health needs from my food support provider	0.04	**0.62**	0.07
2	It is easy for me to get foods which satisfy my cultural needs from my food support provider	0.28	**0.43**	0.05
3	Generally, I get what I need or want from my food support provider	0.07	**0.63**	0.13
4	I am often pleased with the food I get from my food support provider	-0.02	**0.62**	0.07
*5*	*Getting food from my food support provider makes me feel in control*	*0.35*	*0.05*	*0.60*
6	I don’t mind being seen getting food from my food support provider	**0.85**	0.01	-0.02
7	I don’t mind if my family know I get food from my food support provider	**0.41**	0.07	0.28
8	I would rather get food from my food support provider than have to skip meals	-0.15	0.32	**0.57**
9	I feel ashamed when I get food from my food support provider*	**0.78**	-0.35	0.04
10	I feel powerful when I get food from food support provider	**0.82**	-0.12	0.22
*11*	*Getting food from my food support provider is a dignified experience*	*0.44*	*0.40*	*0.21*
*12*	I feel indebted to my food support provider when I get food from them*	*0.20*	*-0.54*	*-0.26*
13	Getting food from my food support provider makes me feel good about myself	**0.61**	0.23	0.17
*14*	*I feel equal to others when I get food from my food support provider*	*0.70*	*0.33*	*-0.17*
15	I have a better quality of life since getting food from my food support provider	-0.02	-0.04	**0.95**
16	I feel like a valued member of society when I get food from my food support provider	**0.82**	0.21	0.03
17	I feel embarrassed when getting food from my food support provider*	**0.82**	-0.02	-0.21
18	I feel taken seriously by the staff at my food support provider	-0.02	**0.93**	-0.05
19	I feel judged when I get food from my food support provider*	**0.46**	0.23	0.05
20	Getting food from my food support provider is a good solution to my problems accessing food	0.28	0.12	**0.56**
21	I look forward to getting food from my food support provider	-0.11	0.28	**0.66**
22	I enjoy interacting with staff at my food support provider	0.17	**0.62**	0.03
23	Getting food from my food support provider is generally a good experience	0.11	**0.69**	0.23

*Item reverse scored in order for inter-correlations to remain positive

Items which were removed are shown in italics. Factor loadings shown in bold highlight which factor onto which the respective item loaded

Subsequently, factor one was made up of eight items, factor two was made up of seven items, and factor three was made up of four items. Items in factor one represented an individual’s own feelings about themselves when accessing food support (for example ‘Getting food from my food support provider makes me feel good about myself’) and about concealing their access to food support to others (for example ‘I don’t mind if my family know I get food from my food support provider’). Therefore, this factor was labelled *‘self-approval and disclosure’*. Items in factor two concerned the actual experience of accessing food support (for example ‘Getting food from my food support provider is generally a good experience’), but also included items referring to how well the support provided met client needs and preferences (for example, ‘Generally, I get what I need or want from my food support provider’), and the interactions with staff at food support service providers (for example ‘I feel taken seriously by the staff at my food support provider’). This factor was thus labelled ‘*dietary and interpersonal satisfaction with food support provider’*. Factor three appeared to be made up of items which described the impact of food support access on the present and future circumstances of individuals (for example ‘I have a better quality of life since getting food from my food support provider’), and was labelled ‘*perceived effectiveness and impact of food support provider’*.

#### Confirmatory factor analysis

**Fig. 3:**
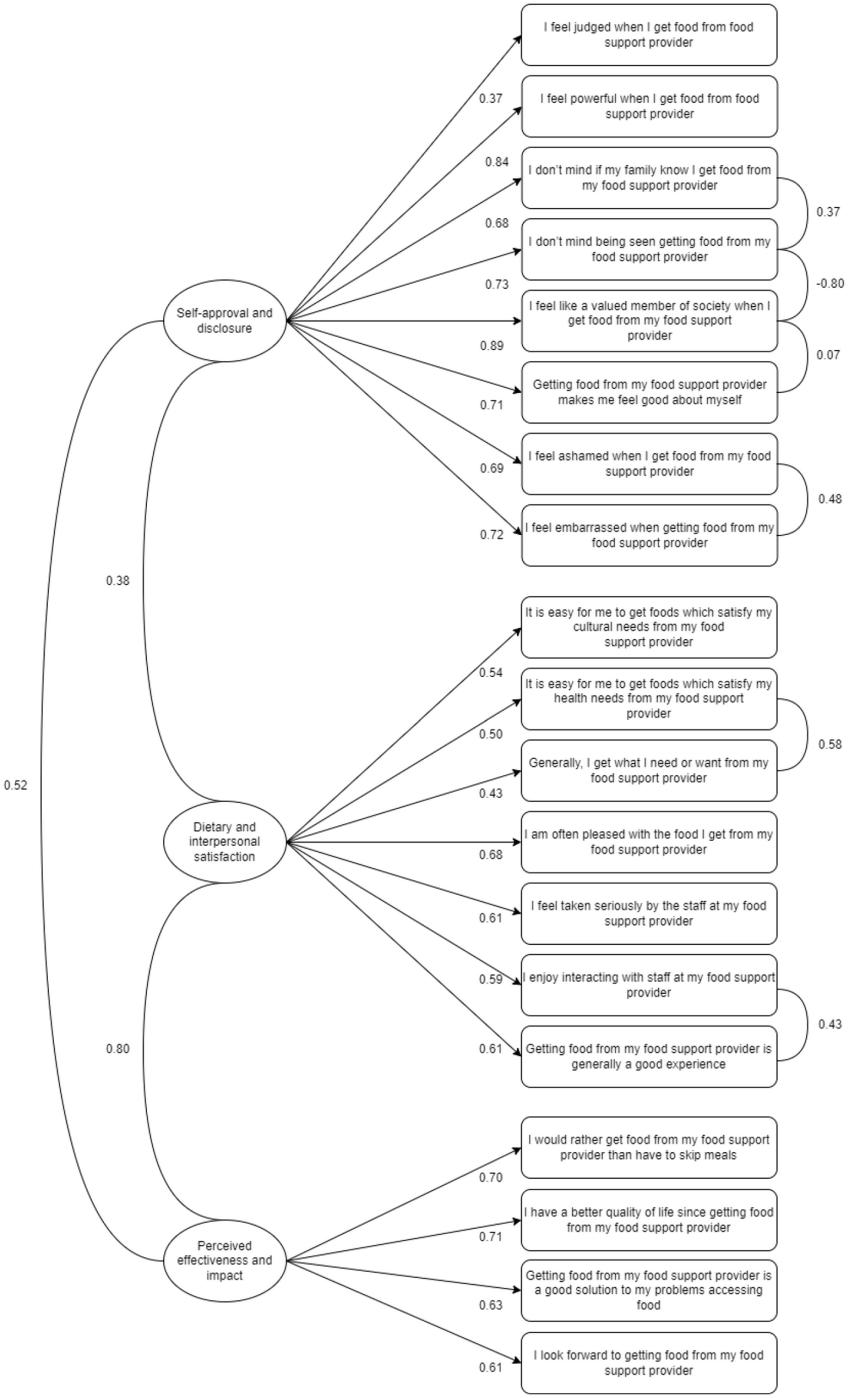
FSES factor model with standardised factor loadings (single-headed arrows) and covariances (double-headed arrows)

To conduct confirmatory factor analysis on the FSES data, the new CFA dataset for the FSES was used (*n* = 59). Initially, confirmatory factor analysis of the three-factor structure suggested a poor fit of the model to the data (CFI = 0.781; TLI = 0.748; RMSEA = 0.114; SRMR = 0.120). Subsequently, covariance pathways based on the modification indices were added to the model (as described in Sect. 2.4.3. above). As a result, the fit of the model was improved yet the resulting model remained an acceptable-to-poor fit (CFI = 0.908; TLI = 0.890; RMSEA = 0.076; SRMR = 0.115). All standardised factor loadings were significant. The resulting 19-item FSES with scoring instructions are provided in the supplementary material. Figure 3 shows the FSES factor model with the standardised factor loadings and covariances. The three factors were all positively correlated, as expected.

#### Internal reliability

The McDonald’s Omega hierarchical value for the full scale was below the acceptable value of 0.7 (w_h_ = 0.53), again indicating that variance in responses produced by a common factor across all items was not reliable. The McDonald’s Omega total value was produced for each subscale and all achieved acceptable estimates (self-approval and disclosure w_t_ = 0.93; dietary and interpersonal satisfaction w_t_ = 0.88; perceived effectiveness and impact w_t_ = 0.84). Therefore, to improve reliability the FSES should be scored as three separate subscales.

#### Convergent validity

Higher scores on the FSES are indicative of a more positive overall experience (including lower levels of self-stigma) when accessing food support. Therefore, it was predicted that scores on the FSES and the RSE scale would be positively correlated, while scores on the FSES and the DASS-21 scale would be negatively correlated. Regressions using latent variables derived from the confirmatory factor analysis, with the corresponding covariance pathways between residuals applied found a positive significant correlation (unstandardised coefficient = 0.384; s.e. = 0.085; 95% CI = 0.217, 0.552) between the factor labelled self-approval and disclosure of the FSES and the RSE, with no other significant correlations found with the other factors. Using scale means instead of latent variables, the overall model (with covariance pathways applied) was significant, explaining 20% of the variance in RSE scores (adjusted R^2^ = 0.20, F(3,119) = 11.39, *p* < 0.001). Again, a positive significant correlation (unstandardised coefficient = 0.396; s.e. = 0.070; 95% CI = 0.258, 0.534; *p* < 0.001) between the factor labelled self-approval and disclosure of the FSES and the RSE, with no other significant correlations found with the other factors.

A significant negative correlation was found between self-approval and disclosure on the FSES and the depression (unstandardised coefficient = -0.356; s.e. = 0.088; 95% CI = -0.528, -0.183), anxiety (unstandardised coefficient = -0.095; s.e. = 0.046; 95% CI = -0.184, -0.005) and stress (unstandardised coefficient = -0.233; s.e. = 0.076; 95% CI = -0.381, -0.085) subscales of the DASS-21 scale, with all other correlations being non-significant. Using scale means as opposed to latent variables, the overall models were all significant (depression: 23% of variance, adjusted R^2^ = 0.226, F(3, 119) = 12.86, *p* < 0.001; anxiety: 7% of variance, adjusted R^2^ = 0.070, F(3, 119) = 4.005, *p* = 0.009; stress: 11% variance, adjusted R^2^ = 0.105, F(3, 119) = 5.772, *p* = 0.001). Regressions using scale means also showed that a significant negative correlation was found between self-approval and disclosure on the FSES and the depression (unstandardised coefficient = -0.358; s.e. = 0.062; 95% CI = -0.482, -0.235 *p* < 0.001), anxiety (unstandardised coefficient = -0.137; s.e. = 0.061; 95% CI = -0.258, -0.016; *p* = 0.026) and stress (unstandardised coefficient = -0.218; s.e. = 0.058; 95% CI = -0.332, -0.104; *p* < 0.001) subscales of the DASS-21 scale, with all other correlations being non-significant.

#### Divergent validity

Using latent variables, no significant correlations were found between the FSES and the IPAQ-SF, indicating good divergent validity of the FSES. Using scale means, the overall model was significant, explaining 14% of variance in IPAQ-SF scores (adjusted R^2^ = 0.14, F [3, 64] = 4.764, *p* = 0.005). Regressions using scale means indicated that IPAQ-SF total scores were significantly correlated to dietary and interpersonal satisfaction scores (unstandardised coefficient = 209.13; s.e. = 90.76, 95% CI = 27.81, 390.45; *p* = 0.025), but no significant associations were found with the other two subscales.

#### Test-retest reliability

Within the two- to nine- week window for retest, data for 13 retest participants were obtained for the FSES, but the data for only eight of these could be linked using the unique ID codes provided in test one and test two. None of the retest cohort were excluded due to high income. Mean scores for the initial test and retest were calculated and test-retest reliability was found to be good (ICC = 0.83) over the few cases obtained.

## Discussion

The present study developed and validated two novel scales which contribute towards understanding the psychological impacts of food insecurity and food support access, focusing on the experience of stigma. The FISS scale aimed to measure concepts of self-stigma when experiencing food insecurity according to Corrigan’s [17, 18, 49] social-cognitive model of internalised stigma (with emphasis on paradoxical empowerment), including stereotype endorsement, non-disclosure, and righteous anger. Meanwhile the FSES scale measures the psycho-social experience, including the experience of self-stigma, of receiving food from a food support provider specifically. Analysis revealed 3-factor structures for both the FISS and FSES scales. Confirmatory factor analysis revealed a good fit of the three-factor model for the FISS scale comprising non-disclosure, righteous anger, and stereotype endorsement subscales, in line with the PaSS-24 scale [48]. Meanwhile, within the FSES scale, acceptable-to-poor fit of the three-factor model was indicated during confirmatory factor analysis, comprising subscales labelled self-approval and disclosure, dietary and interpersonal satisfaction with food support provider, and perceived effectiveness and impact of food support provider. Good test-retest reliability was confirmed across small samples over a two- to nine-week period for both scales.

The development of the FISS and the FSES presents a starting point for measuring stigma and related experiences in the context of food insecurity and accessing food support. In the future, there is potential for these two scales to be used to evaluate interventions to reduce stigma within populations experiencing food insecurity and in working towards a more positive overall experience when accessing food support. Important applications may include: [1] identifying the negative and self-stigmatising aspects of food insecurity or food support access within particular subgroups of the population in order to provide relevant support; and [2] administering the scales both pre and post an intervention which aims to either improve a food support service or address the barriers and issues experienced by individuals identifying as food insecure. Additionally, the FISS and FSES may build upon the existing USDA food insecurity measures by capturing additional dimensions of food insecurity (namely, food insecurity self-stigma and the psychosocial experience (including experiences of self-stigma) when accessing food support), thus enabling examination of the social acceptability of food acquisition. The FISS and FSES measures may also be used alongside USDA measures of food insecurity to explore the associations between stigma experiences and the level of severity of food insecurity.

### Food Insecurity Self-stigma Scale (FISS)

In the development of the FISS scale, items from the PaSS-24 scale [48] were adapted for use within the food insecurity context. The three-factor structure observed in the FISS scale analysis is consistent with the factor structure of the PaSS-24, confirming its applicability to self-stigma relating to food insecurity.

However, in the original PaSS-24 scale all three subscales were shown to be positively correlated with each other. In the present context, while non-disclosure and righteous anger were positively correlated, stereotype endorsement was negatively correlated to both non-disclosure and righteous anger. The negative correlation between stereotype endorsement and righteous anger might suggest that those who experience more feelings of anger regarding their stigmatised position may accept fewer negative stereotypes of individuals experiencing food insecurity. Despite opposing Golay et al.’s [48] theoretical model, this correlation makes logical sense, as individuals who are angrier about the injustice of their stigmatisation may be more likely to refute negative stereotypes about people in their position. In this respect, unlike in the context of mental illness, righteous anger may protect against stereotype endorsement when considering food insecurity status [18, 48]. As such, interventions which aim to empower individuals experiencing food insecurity to acknowledge? the injustice of their stigmatised status and promote righteous anger, while also addressing public misconceptions and presumptuous stereotypes, may be useful in tackling food insecurity stigma.

The RSE and DASS-21 measures were used to assess convergent validity of the FISS scale. It was expected that scores on the FISS scale would be negatively correlated to self-esteem scores on the RSE [71, 72, 84] and positively correlated to scores on the DASS-21 [74, 75]. However, only the subscale non-disclosure significantly correlated with the RSE and the depression, anxiety and stress subscales of the DASS-21, with the correlations being in the expected direction respectively. This indicates that, for individuals experiencing food insecurity, the fear of their status being revealed might be the most salient factor in the development and manifestation of self-stigma, potentially driving lower self-esteem and worsening mental health outcomes. The development of the FISS scale was underpinned by Corrigan’s social-cognitive model of internalised stigma (as per the original PaSS-24 scale) which encompasses the role of paradoxical empowerment through righteous anger [17, 18, 48, 49]. While food insecurity and food support access have been linked to anger and frustration [39, 53–55], Corrigan’s model may not be the most appropriate representation of stigma experiences in the context of food insecurity, and this could therefore explain the lack of convergent validity for the righteous anger and stereotype endorsement subscales. Nonetheless, this study presents a starting point for the measurement of stigma relating to food insecurity and food support access, and future research may seek to apply alternative theoretical models.

The IPAQ-SF was used to assess divergent validity of the FISS scale. The IPAQ-SF measures the levels of physical activity achieved in a day, and can relate to leisure, work and transport. While it was expected that no significant correlations would be found, regressions using latent variables showed that IPAQ-SF scores correlated positively with righteous anger and negatively with non-disclosure (meanwhile, regressions with scale means also showed an association between IPAQ-SF scores and stereotype endorsement). Lower levels of physical activity have been linked to lower mental health status [85–87] which may lead to wanting to conceal food insecurity status in order to protect from further psychological burden. Meanwhile, those who are more physically active may be in a more positive frame of mind, and may be better able to overcome the ‘why try’ effect of low self-efficacy commonly seen in stigmatised groups [84], to embody righteous anger regarding their situation, potentially explaining the positive correlation between IPAQ-SF scores and righteous anger. Further validation of the FISS scale using other concepts to assess divergent validity are recommended.

### Food Support Experiences Scale (FSES)

FSES survey items were developed from the synthesis of qualitative studies summarised within two systematic literature reviews exploring food support access [6, 14]. Overall, the model showed acceptable-to-poor fit, which is likely a result of low power. Repeating the survey over larger samples may increase understanding of the psychometric properties and validity of the FSES. As expected, the three subscales correlated positively, suggesting that the overall experience received at a food support provider might be cumulatively affected by not just how an individual feels about themselves when accessing support, but also how satisfied they are with the food obtained and the interactions with staff, and the level of perceived effectiveness of the support on overall well-being. This supports the idea that access to food support may impact on a broad spectrum of social, physical, and psychological outcomes which influence overall wellbeing [14], and indicates that improving just one of these aspects may aid in creating a more positive and less stigmatising experience for those accessing the support.

Again, the RSE and DASS-21 were used to assess convergent validity. Higher scores on the FSES scale were indicative of a more positive overall experience (including lower levels of self-stigma) when accessing food support, therefore higher FSES scores were expected to be positively associated to RSE score but negatively associated with DASS-21 scores. However, self-approval and disclosure was the only factor which correlated significantly with the RSE and the DASS-21, with associations being in the expected direction. This subscale comprises of items which relate to internalised negative stereotypes of individuals accessing food support and drive to conceal the need to access food support. This association indicates that fear of food insecurity status being revealed might be a salient factor in driving lower self-esteem and poor mental health outcomes not just in people experiencing food insecurity, but also in individuals accessing food support. The lack of significant convergence of RSE and DASS-21 scores with the other two subscales (dietary and interpersonal satisfaction with food support provider and perceived effectiveness and impact of food support provider) may relate to the focus of these subscales being less related to the psychological experience of accessing food support and more related to social, cultural and nutritional experiences. Again, further exploration in larger sample sizes and a reassessment of divergent validity is recommended to ascertain greater validity of the FSES.

### Limitations

Several limitations of the study have been identified. Firstly, as noted previously, difficulties in recruitment demanded a wide recruitment window spanning seven months. As a result, recruitment was partially carried out over the Christmas period, commonly considered a time of financial strain for many, especially those experiencing food insecurity. Additionally, due to the difficulties in recruiting participants, only small samples were obtained for the test-retest, over a long time period. It is possible that during this time, food insecurity status may have changed as a result of financial circumstances or otherwise, and further validation of the two scales would benefit from exploring test-retest in larger samples, over a shorter time period [88].

Recent data from the UK report that 57% of individuals experiencing food insecurity are women [89]. Therefore, women were overrepresented in both the FISS (78.2%) and FSES (78.8%) data. While food insecurity is a highly gendered issue, with women reported to take on the daily burden of lack of adequate access to food within the household [3, 50, 68, 90], future research may explore the validation of the FISS and FSES scales within male populations in order to maximise relevance.

Quantifying stigma within populations experiencing food insecurity is a novel concept with a significant paucity in evidence. The development and validation of the FISS and FSES act as a starting point to exploring the concepts of self-stigma in people experiencing food insecurity, and the psychosocial experience (including self-stigma) of accessing food from a food support provider. Further work is now needed to verify the use of the measures in other populations. For example, the current study’s screening measure for the FSES may have resulted in a skewed focus on food support providers such as food banks, food pantries, community kitchens and hot food providers which are traditionally charitable. Future research could therefore aim to validate the scale in the context of people who are accessing other forms of food support, for example from government food assistance programmes, using measurement invariance analyses. This is important as previous research has indicated the prevalence of (welfare) stigma and negative mental health consequences in this context [91, 92]. Further work may also aim to quantitatively measure other aspects of stigma, including public stigma of people experiencing food insecurity and anticipated stigma of accessing food from a food support provider.

The three-item food insecurity screener used in this study was adapted from the USDA AFSSM [59] by the UK Food Foundation [1] in order to identify individuals experiencing moderate to severe food insecurity. While this was an intentional decision in the current study, use of this measure meant that participants who were experiencing marginal food insecurity or implementing coping strategies to cope with food insecurity were not included. Conducting measurement invariance analyses to explore the relevance of the scales to people experiencing marginal food insecurity is an important future direction, as well as focusing on validating the three-item screener measure. Moreover, we used Corrigan’s theoretical model and the PaSS-24 scale as a basis for the FISS based on theoretical fit and also guided by stakeholder preferences and ethical priorities. However, further quantification of levels of stereotype endorsement and anger in populations experiencing food insecurity compared to people in food security may shed further light on the transferability of this theoretical approach to the food insecurity context. Additionally, the phrasing of the stereotype endorsement subscale in the FISS referred to third parties (e.g. ‘People who have difficulty accessing food are less useful to society’) which may have created issues with the validity of this subscale; an ideal measure of self-stigma would directly assess how a stigmatised person applies negative stereotypes about the condition to themselves. We chose to phrase the items in this way as we were guided by stakeholder preferences and the importance of minimising distress for participants when completing the scale. As stated, the scales developed in this paper provide a first step towards quantification of stigma, and further work to address potential methodological and ethical trade-off is now required.

## Conclusion

This study has developed and validated two novel scales: the Food Insecurity Self-stigma Scale and Food Support Experiences Scale. Their development represents an important first step in measuring self-stigma and related concepts in the context of food insecurity and food support access respectively, as well as the FSES measuring the overall psycho-social experience of accessing food support. Further validation using larger sample sizes and in different populations is now needed Ultimately, the wider use of these scales may have important implications in working towards reducing stigma experiences in the context food insecurity and promoting more positive experiences and better psychological wellbeing for individuals accessing food support.

## Electronic supplementary material

Below is the link to the electronic supplementary material.

Supplementary Material 1

Supplementary Material 2

## Data Availability

The datasets generated and analysed during the current study are available from the corresponding author on reasonable request.The materials generated in the current study are available in the supplementary material and in the Open Science Framework repository (https://osf.io/fmpja).

## Abbreviations

CFA: Confirmatory factor analysis
CFI: Comparative Fit Index
DASS-21: Depression, Anxiety and Stress (21-item) scale
EFA: Exploratory factor analysis
EPR: Experiment Participation Requirement (scheme)
FISS: Food Insecurity Self-stigma Scale
FSES: Food Support Experiences Scale
ICC: Intra-class correlation
IPAQ-SF: International Physical Activity Questionnaire - Short Form
KMO: Kaiser-Meyer-Olkin (statistic)
MLM: Maximum likelihood with Satorra Bentler correction
PaSS-24: Paradox of Self-Stigma scale
RMSEA: Root Mean Square of Approximation
RSE: Rosenberg Self-Esteem (scale)
SRMR: Standardised Root Mean Square Residual
TLI: Tucker-Lewis Index
USDA: United States Department of Agriculture
USDA AFSSM: United States Department of Agriculture Adult Food Security Survey Module
