# Supplementary material for: Towards measuring food insecurity stigma: development and validation of the Food Insecurity Self-stigma Scale and the Food Support Experiences Scale
Source: BMC Public Health. 2024 Nov 30;24:3349. doi: 10.1186/s12889-024-20878-y (PMC11608497; doi:10.1186/s12889-024-20878-y)
Supplement: Supplementary file 1 — Supplementary Material 1 [file 12889_2024_20878_MOESM1_ESM.docx]

| Item |  | Subscale* |
| --- | --- | --- |
| 1 | Because of peoples’ ignorance about how difficult it can be to access enough food, I do not speak to anyone about the problems linked to it | ND |
| 2 | I am really fed up with people's ideas about those who have difficulty accessing enough food | RA |
| 3 | Because of people’s opinions, I do not speak to anybody about having difficulty accessing enough food | ND |
| 4 | The public’s lack of knowledge about issues surrounding accessing enough food makes me angry | RA |
| 5 | People who have difficulty accessing enough food should take on extra work to improve their situation | SE |
| 6 | I try to avoid situations where my difficulty in accessing enough food might be revealed | ND |
| 7 | The stereotypes about people who have difficulty accessing enough food make me angry | RA |
| 8 | Where I can, I avoid talking about the difficulties I have in accessing enough food | ND |
| 9 | The media’s lack of knowledge about the struggles of people who have difficulty accessing enough food is frustrating | RA |
| 10 | People who have difficulty accessing enough food should try to improve their cooking skills to improve their situation | SE |
| 11 | To avoid any prejudice, I choose who I talk to about the difficulties I have accessing enough food | ND |
| 12 | I am angry about the way people who have difficulty accessing enough food are portrayed on television | RA |
| 13 | I do not reveal that I have difficulty accessing enough food to anybody to avoid being judged | ND |
| 14 | Certain people’s attitudes towards people who have difficulty accessing enough food appal me | RA |
| 15 | People who have difficulty accessing enough food should try to improve their budgeting skills to improve their situation | SE |

**Instructions**: The current cost of living crisis is causing many people in the UK to have difficulty accessing enough food. Here is a list of statements dealing with your feelings as a person who may have difficulty accessing enough food for yourself/your family. Please indicate how strongly you agree or disagree with each statement: 1 = strongly disagree; 2 = somewhat disagree; 3 = neither agree nor disagree; 4 = somewhat agree; 5 = strongly agree. There are no right or wrong answers. Do not spend too much time on any statement.

***Subscale**: ND = non-disclosure; RA = righteous anger; SE = stereotype endorsement

**Scoring**: Individual total scores for each subscale should be taken instead of a total score. Higher scores indicate higher amounts of non-disclosure, righteous anger or stereotype endorsement.
