# Supplementary material for: Towards measuring food insecurity stigma: development and validation of the Food Insecurity Self-stigma Scale and the Food Support Experiences Scale
Source: BMC Public Health. 2024 Nov 30;24:3349. doi: 10.1186/s12889-024-20878-y (PMC11608497; doi:10.1186/s12889-024-20878-y)
Supplement: Supplementary file 2 — Supplementary Material 2 [file 12889_2024_20878_MOESM2_ESM.docx]

| Item |  | Subscale* |
| --- | --- | --- |
| 1 | I don’t mind being seen getting food from my food support provider | SAD |
| 2 | I am often pleased with the food I get from my food support provider | DIS |
| 3 | I feel ashamed when I get food from my food support provider* | SAD |
| 4 | It is easy for me to get foods which satisfy my health needs from my food support provider | DIS |
| 5 | I would rather get food from my food support provider than have to skip meals | PEI |
| 6 | Getting food from my food support provider makes me feel good about myself | SAD |
| 7 | I feel taken seriously by the staff at my food support provider | DIS |
| 8 | I feel powerful when I get food from food support provider | SAD |
| 9 | Generally, I get what I need or want from my food support provider | DIS |
| 10 | I have a better quality of life since getting food from my food support provider | PEI |
| 11 | I don’t mind if my family know I get food from my food support provider | SAD |
| 12 | I enjoy interacting with staff at my food support provider | DIS |
| 13 | I feel embarrassed when getting food from my food support provider* | SAD |
| 14 | It is easy for me to get foods which satisfy my cultural needs from my food support provider | DIS |
| 15 | I look forward to getting food from my food support provider | PEI |
| 16 | I feel judged when I get food from my food support provider* | SAD |
| 17 | Getting food from my food support provider is generally a good experience | DIS |
| 18 | I feel like a valued member of society when I get food from my food support provider | SAD |
| 19 | Getting food from my food support provider is a good solution to my problems accessing food | PEI |

**Instructions:** As a person receiving help accessing food, we want to hear about your experiences of receiving this help. The following questions will ask you to think about the times when you access food from your **main** food support provider. Thinking about the help you get from this provider only, please indicate how strongly you agree or disagree with each statement; 1 = strongly disagree; 2 = somewhat disagree; 3 = neither agree nor disagree; 4 = somewhat agree; 5 = strongly agree. There are no right or wrong answers. Do not spend too much time on any statement.

Subscale: PEI = perceived effectiveness and impact of food support provider; DIS = dietary and interpersonal satisfaction with food support provider; SAD = self-approval and disclosure

**Scoring: ***Items 3, 13 and 16 should be reversed scored. Individual scores for each subscale should be taken instead of a total score. Higher scores indicate higher dietary and interpersonal satisfaction, greater perceived impact and effectiveness, or higher levels of self-approval and disclosing regarding food insecurity status (indicates lower levels of self-stigma).
